# Three-Dimensional Evaluation of Skeletal Stability following Surgery-First Orthognathic Approach: Validation of a Simple and Effective Method

**DOI:** 10.1055/a-2058-8108

**Published:** 2023-05-29

**Authors:** Nabil M. Mansour, Mohamed E. Abdelshaheed, Ahmed H. El-Sabbagh, Ahmed M. Bahaa El-Din, Young Chul Kim, Jong-Woo Choi

**Affiliations:** 1 Faculty of Medicine, Department of Plastic and Reconstructive Surgery, Mansoura University, Mansoura, Egypt; 2 Department of Plastic and Reconstructive Surgery, Ulsan University College of Medicine, Seoul Asan Medical Center, Seoul, South Korea

**Keywords:** orthognathic surgery, surgery-first, three-dimensional, 3D evaluation, skeletal stability

## Abstract

**Background** The three-dimensional (3D) evaluation of skeletal stability after orthognathic surgery is a time-consuming and complex procedure. The complexity increases further when evaluating the surgery-first orthognathic approach (SFOA). Herein, we propose and validate a simple time-saving method of 3D analysis using a single software, demonstrating high accuracy and repeatability.

**Methods** This retrospective cohort study included 12 patients with skeletal class 3 malocclusion who underwent bimaxillary surgery without any presurgical orthodontics. Computed tomography (CT)/cone-beam CT images of each patient were obtained at three different time points (preoperation [T0], immediately postoperation [T1], and 1 year after surgery [T2]) and reconstructed into 3D images. After automatic surface-based alignment of the three models based on the anterior cranial base, five easily located anatomical landmarks were defined to each model. A set of angular and linear measurements were automatically calculated and used to define the amount of movement (T1–T0) and the amount of relapse (T2–T1). To evaluate the reproducibility, two independent observers processed all the cases, One of them repeated the steps after 2 weeks to assess intraobserver variability. Intraclass correlation coefficients (ICCs) were calculated at a 95% confidence interval. Time required for evaluating each case was recorded.

**Results** Both the intra- and interobserver variability showed high ICC values (more than 0.95) with low measurement variations (mean linear variations: 0.18 mm; mean angular variations: 0.25 degree). Time needed for the evaluation process ranged from 3 to 5 minutes.

**Conclusion** This approach is time-saving, semiautomatic, and easy to learn and can be used to effectively evaluate stability after SFOA.

## Introduction

The surgery-first orthognathic approach (SFOA) has many advantages. It decreases the total treatment time since the lengthy presurgical orthodontic stage is skipped. It also provides immediate improvement in patients' aesthetic profile, facilitates dental movements in the postsurgical phase by the regional acceleratory phenomenon, and improves patients' quality of life. 1 2 3 4 5

The only debate preventing widespread acceptance of SFOA is postsurgical stability. While many consider it as stable as the conventional approach, 6 7 other studies associated it with greater postsurgical mandibular relapse. 8 Long-term skeletal stability after orthognathic surgery (percentage of treatment change that was retained after 1 year or longer [achieved total movement − amount of relapse/total movement]) is affected by numerous factors, including surgical technique, temporomandibular joint and condyle seating, muscle and soft tissue effects, and dental movements 9 : thus, measuring postoperative movements and relapses is crucial for assessing the outcomes of the used approach as well as to delineate the effects of different factors on the surgical outcomes.

Two-dimensional (2D) cephalometry provides reasonable information about vertical and sagittal bone movements as well as rotations around the x-axis (pitch); however, it fails to comprehend horizontal (side to side) movements or rotations around the y- and z-axes (yaw and roll). Moreover, its results can be easily confounded by image overlap, landmark identification, and the experience of the observer. 10 Several researchers have reported a significant difference between 2D and 3D cephalometric measurements. 11 12

In contrast, 3D evaluation can accurately assess postsurgical stability. However, most of the methods in the literature are difficult to replicate, especially for surgeons. These methods depend on the reidentification of a large number of cephalometric landmarks on postoperative images, measurements of each point with the three planes of space, a process which accumulates identification errors leading to inaccuracies, 13 14 or an automatic image registration process, which is time-consuming and requires at least one additional software other than the one used in planning. 15 16 Thus, 3D evaluation is not yet universally applied.

Rapid and extensive dental movements after surgery are another issue encountered when evaluating the 3D stability of SFOA. Relying on dental landmarks, as suggested by other studies, 13 15 17 will not provide accurate information on the skeletal movement. Moreover, a separate 3D segmentation of the maxilla and mandible and dental superimposition by a 3D dental scan are required to avoid the artifacts caused by dental braces during postsurgical orthodontics. This makes the virtual evaluation process more time-consuming.

In a systematic review, Gaber et al 14 suggested three criteria for accurate 3D evaluation of orthognathic surgery planning and stability: “1) an automatic voxel-based registration based on the cranial base; 2) Automated or semiautomated evaluation of the outcome indicative of changes in 3D—whether it is translational or rotational, based on the different axes (x, y, z); 3) Inter- and intra-observer reliability should be used to validate the results.” 14 In another study by Almukhtar et al 18 for 3D assessment of surgical changes following orthognathic surgery, no significant difference was found between surface- and voxel-based registration methods.

The purpose of the present study was to propose and validate a simple, time-saving, semiautomatic, and accurate method for 3D evaluation of orthognathic surgery that could be applied to SFOA. This method meets the criteria suggested by Gaber et al, 14 and is hypothesized to have excellent reliability if the intraclass correlation coefficient (ICC) between intra- and interobserver measurements is excellent (ICC > 0.95). 19

## Methods

This retrospective study included patients with class 3 malocclusion who underwent bimaxillary surgery (Le Fort I and bilateral sagittal split osteotomy) without any presurgical orthodontic work-up, between 2020 and 2021, with accessibility to a good quality computed tomography (CT) or cone-beam computed tomography (CBCT) scans of the head region at three different time points: preoperatively (T0), immediately postoperatively (T1), and at least 1 year after surgery (T2); all scans were captured with the condyles and occlusion seated in centric relation. Exclusion criteria were cleft lip/palate, congenital anomalies, and posttraumatic and temporomandibular joint pathology. The study was approved by the institutional review board (registration number: S2022-2275-0001). The aims of this study were as follows: (1) to describe a simple, time-saving, and effective method for evaluation of postoperative stability following orthognathic surgery; (2) to validate this proposed method by calculating the ICC between the repeated measurements of two independent observers; and (3) to define a novel set of linear and angular measurements that could represent the maxillary and mandibular movements after SFOA using the lowest number of points while avoiding dental artifacts and postsurgical movements. A written informed consent was obtained from the patient for illustrative figures and videos.

### Data Acquisition and Software Settings

CT or CBCT images were exported into the Digital Imaging and Communications in Medicine (DICOM) format. The DICOM files of each stage (T0, T1, T2) were imported into PROPLAN CMF v 3.0.0 software (Materialise, Leuven, Belgium) and segmented using bone threshold within the program to form a 3D virtual image of the head skeleton. Each of the three images (T0, T1, and T2) was assigned a different contrasting color for better identification in the alignment and landmarking stages.

For automatization of the measurement process, a set of angles and distances ( Table 1 ) were predefined in the program. This was done by using the cephalometry wizard within the software or directly by importing an extensible markup language (XML) file, which we developed for this evaluation process ( Supplementary Material , available in online version only).

**Table 1:** Landmarks and measurements for each model (T0, T1, T2)

Landmarks	
Anterior nasal spine (ANS)	The most anterior midpoint of the anterior nasal spine of the maxilla
Posterior nasal spine (PNS)	The most posterior midpoint of the posterior nasal spine of the palatine bone
Point (A)	The point of maximum concavity in the midline of the alveolar process of the maxilla
Right mental (rt M)	Center of the right mental foramen of the mandible
Left mental (lt M)	Center of the left mental foramen of the mandible
Lines (automatically generated)	
Mental line	Line connecting rt M and lt M
ANS to A line	Line connecting ANS to point A
Basion to midmental	Line connecting basion to middle of mental line
Basion to ANS	Line connecting basion to ANS
Distances (automatically calculated)	
ANS (coronal, sagittal, and horizontal)	ANS to coronal plane ANS to sagittal plane ANS to horizontal plane
PNS (coronal, sagittal, and horizontal)	PNS to coronal plane PNS to sagittal plane PNS to horizontal plane
Rt M (coronal, sagittal, and horizontal)	Rt M to coronal plane Rt M to sagittal plane Rt M to horizontal plane
Lt M (coronal, sagittal, and horizontal)	Lt M to coronal plane Lt M to sagittal plane Lt M to horizontal plane
Angles (automatically calculated; Fig.4)	
Mandibular roll	Angle between mental line and sagittal plane
Mandibular pitch	Angle between basion to midmental line and horizontal plane
Mandibular yaw	Angle between mental line and coronal plane
Maxillary roll	Angle between ANS to A line and sagittal plane
Maxilla pitch	Angle between basion to ANS line and horizontal plane
Maxilla yaw	Angle between basion to ANS line and sagittal plane

### Alignment



**Video 1** showing the process of alignment.


**Fig. 1:**
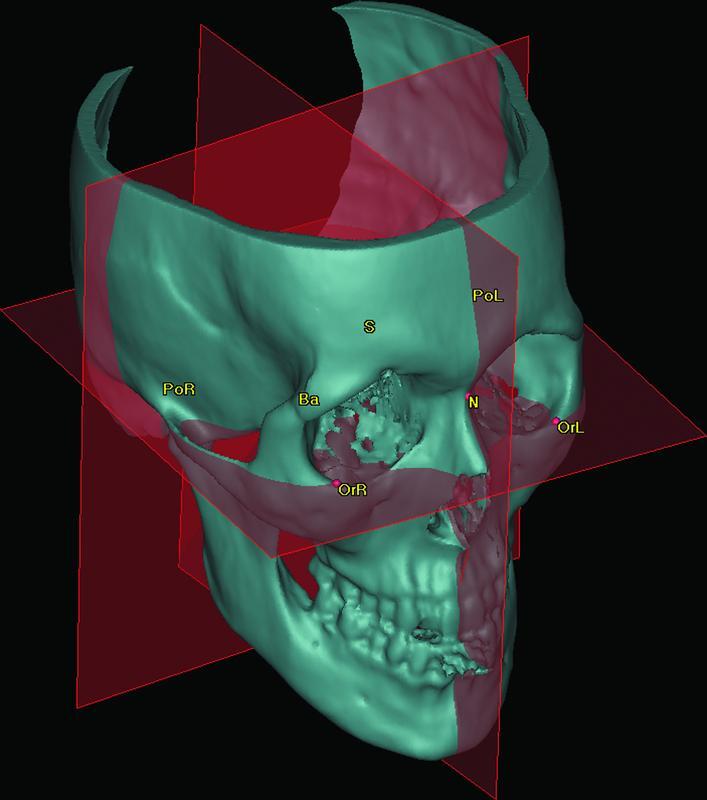
Landmarks used for construction of orientation planes. Ba, basion; N, nasion; OrL, left orbitale; OrR, right orbitale; Pol, left porion; PoR, right porion; S, sella.

Virtual 3D image (T0) was set as the base model, to which T1 and T2 were aligned. For orientation and measurements, seven landmarks are identified on the T0 image, and subsequently, the program automatically defined three planes of space (horizontal, sagittal, and coronal) as described in Table 2 ( Fig. 1 ).

**Table 2:** T0 orientation landmarks and planes

	**Definition**
Landmark	
Orbitale (Or) right and left	The most inferior point of each infraorbital rim
Porion (Po) right and left	The most superior point of each external acoustic meatus
Basion (Ba)	The most anterior point of the great foramen (foramen magnum)
Nasion (N)	The midpoint of the frontonasal sutures
Sella (S)	The center of the hypophyseal fossa (sella turcica)
Planes (automatically defined)	
Horizontal plane (Frankfurt)	Right and left orbitale, right and left porion
Midsagittal plane	Nasion, basion, and sella
Coronal plane	Perpendicular to horizontal and midsagittal planes through basion

**Fig. 2:**
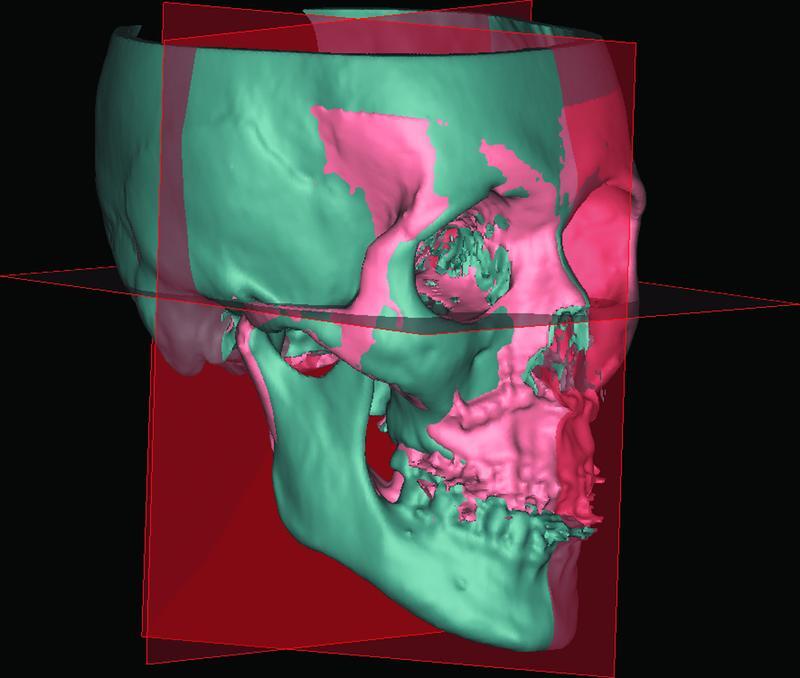
Postoperative model T1 (red) aligned to preoperative model T0 (gray) based on the fixed anterior cranial base.

T2 and T3 virtual models were subsequently aligned to T0 using an automatic functionality of the program. To ensure that the program uses the fixed cranial base (not the osteotomized maxilla and mandible), we marked the external surfaces of both superior orbital ridges and both zygomatic arches ( Fig. 2 ). Subsequently, a quick and accurate automatic surface-based alignment was made ( Video 1 ).

### Landmark Identification



**Video 2** showing the process of landmarking.


**Fig. 3:**
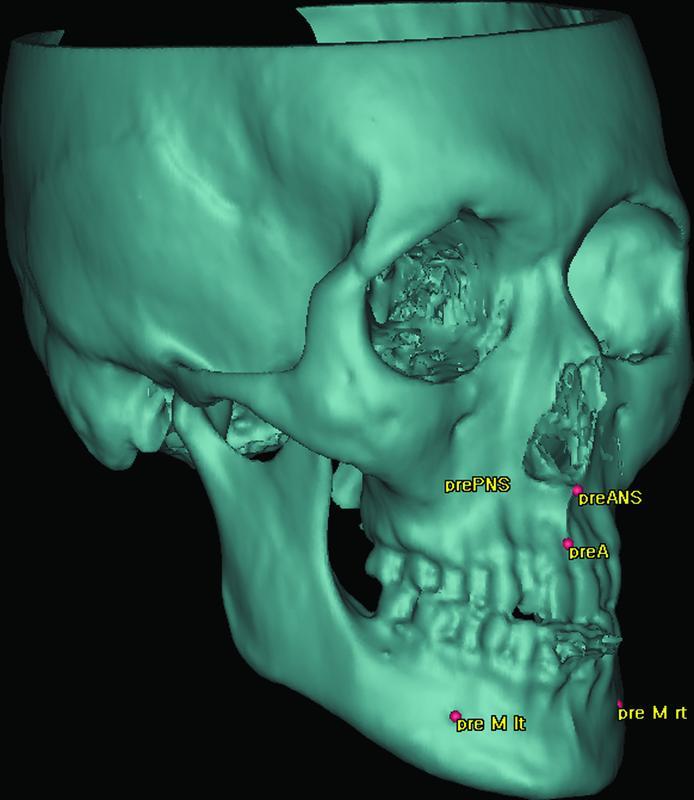
Five cephalometric landmarks used in the evaluation process defined to the preoperative model T0. preA, point (A) of T0; preANS, anterior nasal spine of T0; prePNS, posterior nasal spine of T0; pre M lt, left mental of T0; pre M rt, right mental of T0.

Five sharp anatomical landmarks ( Fig. 3 ) were defined on each virtual model separately (T0, T1, and T2), three on the maxilla (anterior nasal spine [ANS], posterior nasal spine [PNS], point A) and two on the mandible (right and left mental foramina [rt M and lt M]) ( Video 2 ).

### Measurements and Recordings

**Fig. 4:**
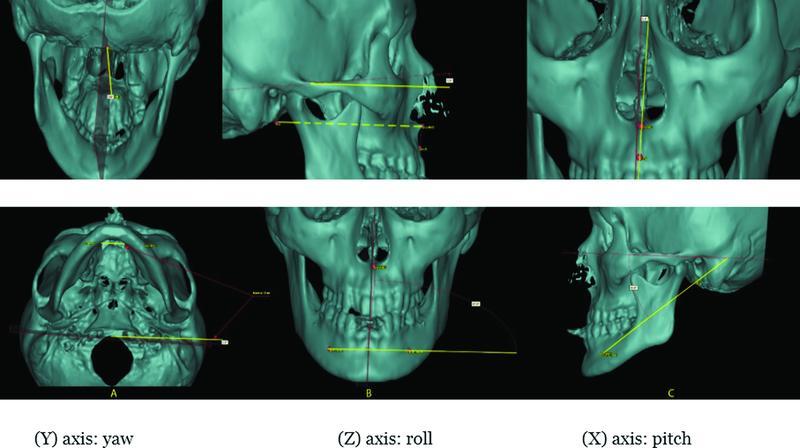
New angles measuring rotational changes: first row shows maxillary angles, and second row shows mandibular angles. Column A: angles used to measure yaw rotation; column B: angles used to measure roll rotation; column C: angles used to measure pitch rotation.

Through an automatic process, the program measured the distances between the landmarks (ANS, PNS, rt M, and lt M) on each virtual model and the three planes of space. The three rotational movements (roll, pitch, and yaw) for the maxilla and mandible of each model were also measured separately by using the predefined angles ( Table 2 ; Fig. 4 ).

All measurements were exported in a spreadsheet format (MS Excel, Microsoft Corporation).

For each case, the time elapsed from the start of the alignment process till the measurements was recorded in seconds.

### Validation

To validate the current method and to evaluate the accuracy of measurements, two independent observers performed the alignment and landmarking for all cases. One of them repeated the same steps after 2 weeks; thereafter, all the measurements were automatically calculated and recorded.

### Statistical Analyses

Measurements were expressed as mean absolute differences (MADs), mean and standard deviation (SD) of the surgical movements (T1–T0), and surgical relapse (T2–T1), for both translational and rotational changes.

ICCs (absolute agreement in a two-way mixed-effects model) were used to test the repeatability of measurements by comparing inter- and intraobserver measurements. ICCs ˃ 0.80 and ˃ 0.95 were defined as good and excellent, respectively.

The mean linear movements of both mental foramina landmarks (rt M and lt M) in relation to the three planes defined the mean mandibular translational movement. In addition, the mean movements of the ANS and PNS landmarks defined the mean maxillary translational movements.

All statistical analyses were performed using IBM SPSS Statistics 20 (IBM Corporation, Armonk, NY).

## Results

The study enrolled 12 patients (8 females and 4 males) whose mean age at surgery was 20 years (range, 16–25 years).

### Duration of the Evaluation Process

The mean time for the process of alignment and landmarking of all three virtual models was 3 minutes 40 seconds (±30 seconds) for the first observer and 4 minutes 10 seconds (±50 seconds) for the second observer.

### Validation of the Method

The MADs, SD, and ICCs for intra- and interobserver variations for the maxillary and mandibular measurements are displayed in Tables 3 and 4 , respectively.

**Table 3:** Intra- and interobserver intraclass correlation coefficients (ICCs), mean absolute differences (MAD), and standard deviations (SD) for translational and rotational measurements of the maxilla in both surgical movements (T0–T1) and postsurgical relapse (T1–T2)

			Rotational			Translational		
			Yaw	Pitch	Roll	Anterior/posterior	Side to side	Up/down
Accuracy of surgical measurements (T1–T2)	Intraobserver	MAD	0.04	0.06	0.04	0.05	0.02	0.03
		SD	0.05	0.07	0.05	0.06	0.05	0.04
		ICC	1	0.99	0.99	0.99	1	1
	Interobserver	MAD	0.24	0.25	0.24	0.16	0.16	0.16
		SD	0.05	0.06	0.05	0.05	0.05	0.05
		ICC	0.99	0.98	0.95	0.99	0.99	0.99
Accuracy of postsurgical relapse measurements	Intraobserver	MAD	0.04	0.05	0.04	0.03	0.01	0.03
		SD	0.05	0.05	0.05	0.05	0.03	0.04
		ICC	0.99	0.99	0.99	1	0.99	0.99
	Interobserver	MAD	0.14	0.14	0.16	0.16	0.16	0.16
		SD	0.06	0.06	0.08	0.05	0.05	0.05
		ICC	0.99	0.98	0.95	0.99	0.98	0.98

Abbreviations: ICC, intraclass correlation coefficient; MAD, mean absolute difference; SD, standard deviation.

**Table 4:** Intra- and interobserver intraclass correlation coefficients (ICCs), mean absolute differences (MAD), and standard deviations (SD) for translational and rotational measurements of the mandible in both surgical movements (T0–T1) and postsurgical relapse (T1–T2)

			Rotational			Translational		
			Yaw	Pitch	Roll	Anterior/posterior	Side to side	Up/down
Accuracy of surgical measurements (T1–T2)	Intraobserver	MAD	0.05	0.04	0.03	0.02	0.04	0.03
		SD	0.05	0.05	0.05	0.04	0.06	0.05
		ICC	1	1	0.99	1	1	1
	Interobserver	MAD	0.25	0.24	0.23	0.17	0.16	0.18
		SD	0.05	0.05	0.05	0.04	0.05	0.05
		ICC	0.99	0.99	0.99	0.99	1	0.99
Accuracy of postsurgical relapse measurements	Intra-observer	MAD	0.03	0.02	0.03	0.06	0.03	0.04
		SD	0.05	0.04	0.05	0.05	0.05	0.05
		ICC	1	1	0.99	1	0.99	0.99
	Interobserver	MAD	0.16	0.17	0.16	0.17	0.18	0.17
		SD	0.05	0.04	0.05	0.04	0.05	0.05
		ICC	0.99	0.99	0.98	0.99	0.99	0.99

Abbreviations: ICC, intraclass correlation coefficient; MAD, mean absolute difference; SD, standard deviation.

None of the mean intraobserver variations exceeded 0.06 mm for linear measurements with ICCs between 0.99 and 1. The highest interobserver MAD was 0.18 mm (SD = 0.05) for linear movements and 0.25 degrees for angular movements (SD = 0.06), with ICCs (0.95–1) demonstrating a very high repeatability between the measurements.

**Fig. 5:**
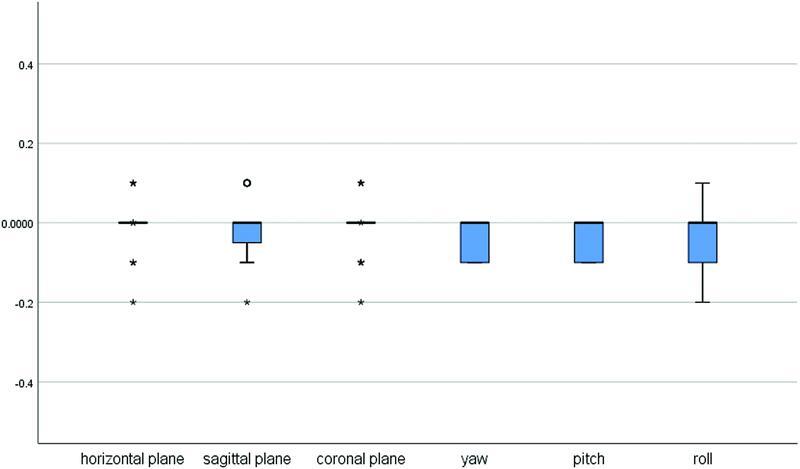
Boxplot showing the differences in intraobserver measurements in the three translational and three rotational movements for (T1–T0) and (T2–T1).

**Fig. 6:**
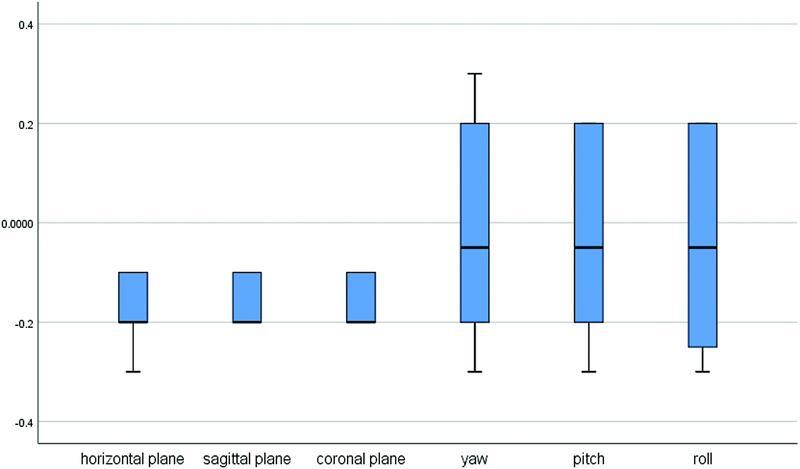
Boxplot showing the differences in interobserver measurements in the three translational and three rotational movements for (T1–T0) and (T2–T1).

The level of agreement between intra- and interobserver measurements is presented using boxplots in Figs. 5 and 6 , respectively. The differences were close to zero in intraobserver measurements and not exceeding 0.3 for interobserver differences. Linear intraobserver measurements were subjected to the least observer-dependent errors followed by the angular intraobserver measurements, while the angular interobserver measurements showed the greatest variations.

### 3D Skeletal Relapse Assessment following SFOA

Since there were no significant differences in the measurements of the two observers, the first observer recordings were used to assess the relapse.

The means and SDs of all landmarks' linear movements in relation to the three planes, as well as maxillary and mandibular rotational movements for both (T1–T0) and (T2–T1), are displayed in Tables 5 and 6 , respectively.

**Table 5:** Means and standard deviations of linear and rotational movements (T1–T0)

Mean		SD
Distance to Frankfurt horizontal plane (−: upward; +: downward)		
ANS	**−** 0.8833	2.03552
PNS	**−** 4.1167	2.03060
Right mental	**−** 3.4083	2.79527
Left mental	**−** 3.0500	1.90096
Distance to midsagittal plane (+: right side; **−** : left side)		
ANS	0.97	1.8
PNS	0.40	0.95330
Right mental	1.08	4.2
Left mental	1.14	4.06
Distance to coronal plane (+: anterior; −: posterior)		
ANS	2.4167	1.50625
PNS	2.7417	1.46502
Right mental	**−** 6.6083	3.44659
Left mental	**−** 6.2167	2.88313
Maxilla rotational changes (+: counterclockwise; −: clockwise) in relation to the axis		
Roll	**−** 6.3	2.1
Pitch	1.2	1.6
Yaw	0.52	1.2
Mandible rotational changes (+: counterclockwise; −: clockwise) in relation to the axis		
Roll	**−** 2.03	4.26
Pitch	**−** 2.19	1.50
Yaw	0.56	1.94

Abbreviations: ANS, anterior nasal spine; PNS, posterior nasal spine; SD, standard deviation.

Note: Distance to midsagittal plane was calculated in absolute mean to avoid errors that occur when crossing the midline (+ to right side, − to left side).

**Table 6:** Means and standard deviations of linear and rotational movements of postsurgical relapse (T2–T1)

	Mean	SD
Distance to Frankfurt horizontal plane (−: upward; +: downward)		
ANS	−0.0583	0.80166
PNS	0.1750	0.67572
Right mental	−1.0417	1.58370
Left mental	−1.4583	1.21988
Distance to midsagittal plane (+: right side; **−** : left side)		
ANS	0.09	0.64
PNS	−0.9	0.62
Right mental	−0.23	1.20
Left mental	0.32	0.97
Distance to coronal plane (+: anterior; **−** : posterior)		
ANS	−1.9583	1.21988
PNS	−0.1167	1.23571
Right mental	2.5583	2.35891
Left mental	2.5667	2.11803
Maxilla rotational changes (+: counterclockwise; **−** : clockwise) in relation to the axis		
Roll	0.41	1.85
Pitch	−0.59	0.78
Yaw	0.05	0.65
Mandible rotational changes (+: counterclockwise; −: clockwise) in relation to the axis		
Roll	1.09	1.47
Pitch	1.55	1.34
Yaw	−1.3	1.13

Abbreviations: ANS, anterior nasal spine; PNS, posterior nasal spine; SD, standard deviation.

Note: Distance to midsagittal plane was calculated in absolute mean to avoid errors that occur when crossing the midline (+: to right side; −: to left side).

The mean linear movements of the mandible and maxilla are displayed in Table 7 , which shows the greatest degree of mandibular relapse in the sagittal movements (2.5 ± 2 mm), while the maxilla was relatively stable 1 year after surgery with the greatest mean relapse of 0.7 mm in the posterior direction.

**Table 7:** Mean linear movements and surgical relapse of the mandible and maxilla

	Surgical movement	Surgical relapse
Mandible		
Vertical	Upward (3.22 ± 2)	Upward (1.2 ± 1.2)
Horizontal	Right side (1.11 ± 4.04)	Right side (0.6 ± 1.4)
Sagittal	Posterior (6.4 ± 3)	Anterior (2.5 ± 2)
Maxilla		
Vertical	Upward (2.5 ± 1.4)	Downward (0.03 ± 0.4)
Horizontal	Right side (0.6 ± 1.4)	Left side (0.04 ± 1.11)
Sagittal	Anterior (2.2 ± 1.4)	Posterior (0.7 ± 1)

## Discussion

Measuring skeletal stability after orthognathic surgery is a very critical step in evaluating the achieved long-term aesthetic and functional results, testing the reliability of the surgical-orthodontic approach, and studying factors affecting the amount of postoperative skeletal relapse. 2D cephalometric studies have long been used for these purposes. However, they cannot provide enough information to assess the amount of horizontal relapse (side-to-side movements) as well as the degree of pitch and yaw rotations. On the other hand, 3D evaluations described in the literature are rather complicated approaches that consume a lot of time and include the application of numerous steps. In this study, we described and validated a simple yet effective method for the 3D evaluation of skeletal stability after orthognathic surgery that could be applied effectively to SFOA. We compared intra- and interobserver measurements of two independent observers, where the difference between the repeated measurements was used to evaluate repeatability. Minimal differences with repetition indicated a small margin of error in the essential steps (alignment and landmarking).

The results demonstrated the reliability of this new method as shown by the excellent correlation coefficient range and low variations in the repeated measurements. The linear differences were below 0.3 mm, which is less than the clinically acceptable threshold of repeatability of 0.5 mm proposed in previous studies. 13 16 20 This high accuracy could be attributed to the following:

- The alignment process was automatic, skull parts not affected by surgery (zygomatic arches and superior orbital ridges).
- All the linear measurements depended on sharp anatomical landmarks (ANS, PNS, rt M, and lt M), which are easily identifiable on the 3D skeletal virtual model, except for point A on the maxilla, which was used only for angular measurements.
- The reference planes (horizontal, sagittal, and coronal) were defined one time only on T1 before the alignment of T2 and T3. This makes all the linear and angular measurements to be dependent only on the five landmarks, unaffected by the orientation planes even if they contain errors.
- All measurements and recordings were obtained automatically, using a single software, which prevented all potential human errors.

The key advantage of this new approach is that it saves time, with a range of 3 to 5 minutes for the entire process from aligning the three virtual models to measurements. To the best of our knowledge, with the same level of accuracy reported in the literature, 17 this is the least time-consuming 3D method of evaluating stability in orthognathic surgery, compared with similar studies, which either failed to provide the time duration of the assessment procedure or were considered to be time consuming. Nada et al 16 reported 30 to 40 minutes per set of two CBCT scans, and Shaheen et al 15 reported a mean time of 20 minutes for two modules. Automatization of alignment and measurements as well as dependence on only five landmarks makes the procedure less time-consuming.

The small number of landmarks also increases the accuracy of measurements. The range of ICCs for mandibular angular measurements (0.98–0.99) was better than those for the maxilla (0.95–1); however, both measurements indicated excellent correlations. The minor difference could be attributed to using three landmarks (ANS, PNS, point A) in assessing maxillary rotations, compared with only two landmarks for the mandible. This finding supports the idea that increasing cephalometric landmarking is associated with an increase in measurement errors and variations with repetition. 21 22

Another advantage of this method is using a single software, which is commonly used in segmentation and surgery planning. This improves the surgeon's familiarity with the software leading to faster and easy assessment. It is also possible to apply this method and its associated measurements ( Supplementary Material , available in online version only) to any other planning software, which will make the process of evaluation comparatively easy and effective. Previous studies reported the use of at least two software for evaluation. 13 17 Yet another study used one software for evaluation and the other for planning and segmentation, 15 which increases the complexity and time consumption of the procedure.

This novel method of evaluation is independent of dental landmarks, making it more suitable for SFOA, which is characterized by major dental movements after surgery. Besides, when we rely on dental landmarks for evaluation, there is a need to superimpose scanned dental surfaces to the maxilla and mandible separately to avoid artefacts of dental braces in postoperative CT. The superimposition process requires segmentation of the mandible and maxilla separately, which contributes to an increase in the time required and inaccuracy of the process. 23

Findings of 3D evaluation of skeletal stability after SFOA show high stability of the maxilla postsurgery. In the case of the mandible, there was a mean forward relapse of 2.5 mm (±2), which is consistent with previous findings. 7 19 24 25 There was also an upward vertical relapse of 1.2 mm (±1.2). This vertical reduction in the SFOA can be explained by a more vertical bite settling after surgery, especially in cases with severe occlusal interference between arches. 26 Moreover, there were side-to-side horizontal mandibular relapses of 2 mm (±1.8). These significant horizontal changes could not be recorded by using conventional 2D imaging, indicating the importance of 3D evaluation in orthognathic surgery.

The presented method had a few limitations. The software used cannot detect subdecimeter (<0.1 mm) measurements. In addition, the angles described defining rotational movements of the maxilla and mandible are novel and nonstandardized, which can only be compared with previous readings of the same angles. Hence, the differences between them do not express the true magnitude of movement.

This study presents a novel method of 3D evaluation of skeletal stability after orthognathic surgery. This method is semiautomatic, time-saving, and easy to learn and could be used effectively to measure stability after SFOA. Only five anatomical landmarks were used to assess the stability of the maxilla and mandible. Inter- and intraobserver measurements proved a high reliability and accuracy of these measurements.
